# Implications Enzymatic Degradation of the Endothelial Glycocalyx on the Microvascular Hemodynamics and the Arteriolar Red Cell Free Layer of the Rat Cremaster Muscle

**DOI:** 10.3389/fphys.2018.00168

**Published:** 2018-03-16

**Authors:** Ozlem Yalcin, Vivek P. Jani, Paul C. Johnson, Pedro Cabrales

**Affiliations:** ^1^Koç University School of Medicine, Sariyer, Istanbul, Turkey; ^2^Department of Bioengineering, University of California, San Diego, San Diego, La Jolla, CA, United States

**Keywords:** cell free layer, endothelial glycocalyx, endothelial surface layer, plasma layer, enzymatic degradation, blood flow, shear stress, microcirculation

## Abstract

The endothelial glycocalyx is a complex network of glycoproteins, proteoglycans, and glycosaminoglycans; it lines the vascular endothelial cells facing the lumen of blood vessels forming the endothelial glycocalyx layer (EGL). This study aims to investigate the microvascular hemodynamics implications of the EGL by quantifying changes in blood flow hydrodynamics post-enzymatic degradation of the glycocalyx layer. High-speed intravital microscopy videos of small arteries (around 35 μm) of the rat cremaster muscle were recorded at various time points after enzymatic degradation of the EGL. The thickness of the cell free layer (CFL), blood flow velocity profiles, and volumetric flow rates were quantified. Hydrodynamic effects of the presence of the EGL were observed in the differences between the thickness of CFL in microvessels with an intact EGL and glass tubes of similar diameters. Maximal changes in the thickness of CFL were observed 40 min post-enzymatic degradation of the EGL. Analysis of the frequency distribution of the thickness of CFL allows for estimation of the thickness of the endothelial surface layer (ESL), the plasma layer, and the glycocalyx. Peak flow, maximum velocity, and mean velocity were found to statistically increase by 24, 27, and 25%, respectively, after enzymatic degradation of the glycocalyx. The change in peak-to-peak maximum velocity and mean velocity were found to statistically increase by 39 and 32%, respectively, after 40 min post-enzymatic degradation of the EGL. The bluntness of blood flow velocity profiles was found to be reduced post-degradation of the EGL, as the exclusion volume occupied by the EGL increased the effective volume impermeable to RBCs in microvessels. This study presents the effects of the EGL on microvascular hemodynamics. Enzymatic degradation of the EGL resulted in a decrease in the thickness of CFL, an increase in blood velocity, blood flow, and decrease of the bluntness of the blood flow velocity profile in small arterioles. In summary, the EGL functions as a molecular sieve to solute transport and as a lubrication layer to protect the endothelium from red blood cell (RBC) motion near the vessel wall, determining wall shear stress.

## Introduction

Blood is a multiphase fluid, consisting of red blood cells (RBCs), plasma, proteins, and electrolyte, with nearly 45% of blood volume occupied by RBCs. Consequently, the resistance to blood flow and blood apparent viscosity are largely dependent on the shear stress experienced by RBCs (Zhang et al., 2009). Hydrodynamic forces during blood flow force RBC migration away from the vessel wall, generating a RBC depleted zone near the vessel wall, or cell free layer (CFL), and a RBC packed core near the center of the blood vessel (Cokelet and Goldsmith, 1991). The formation of the CFL in flowing blood is an important hemodynamic feature of blood flow in the microcirculation. The CFL corresponds to a low viscosity zone, which acts as a lubricating hydrodynamic layer, reducing resistance to flow between the central core of RBCs and the vessel wall (Reinke et al., 1987; Zhang et al., 2009). The thickness of the CFL is proportional to the yield stress of the flowing fluid and depends on the hematocrit (Hct), RBC aggregation, vessel geometry, the migration forces driving the RBCs to away from the vessel wall, and the endothelial glycocalyx.

The endothelial cells lining the luminal side of blood vessels are covered with membrane-bound macromolecules, glycoproteins, and proteoglycans known as the endothelial glycocalyx. The polyanionic nature of the glycocalyx is a consequence of the glycoproteins, bearing acidic oligoasaccharides and terminal sialic acids (SAs) and the proteoglycans, with their glycosaminoglycan (GAG) side chains, that compose it. As such, under physiological conditions the glycocalyx is negatively charged. Association of the polyanionic components of the endothelial glycocalyx layer (EGL) with blood-borne molecules results in an extended endothelial surface layer (ESL) that arises from the EGL (Adamson and Clough, 1992; Pries et al., 2000). Enzymes and their inhibitors, growth factors, plasma proteins, cytokines, cations, and water, all associate with this matrix of biopolyelectrolytes (Bernfield et al., 1999; Osterloh et al., 2002). In addition to glycoproteins, proteoglycans and GAGs, the EGL mainly consists of keratan sulfate, chondroitin sulfate, dermatan sulfate, heparan sulfate, and hyaluronan. The EGL provides selective vasoprotective barrier properties of the vascular wall against vascular leakage, platelet, and leukocyte adhesion (Nieuwdorp et al., 2005). In intact blood vessels, the glycocalyx has an important role in a wide range of physiological processes including increasing vascular permeability, sensing shear stress, and impeding leukocyte and platelet adhesion to the vessel wall (Nieuwdorp et al., 2005; Weinbaum et al., 2007). *In vivo* observations have reported a structure named the ESL, which includes the glycocalyx and other plasma proteins attached to the glycocalyx (Pries et al., 2000). The ESL thickness has been reported to be approximately range from 0.5 μm to over 1 μm (Weinbaum et al., 2007). In general, reports suggest that the ESL thickness varies in different regions of the vascular network (Becker et al., 2010). It is well known that an ESL exists but *in vivo* thickness of the layer is controversial because of the different values from the different techniques in the literature.

This study was conducted to evaluate the effect of the EGL on microvascular blood flow in small arterioles using the rat cremaster muscle preparation. The endothelial glycocalyx was enzymatically degraded by enzymatic degradation via systemic infusion of enzymes to cleave specific GAGs (heparinase, chondroitinase, and hyaluronidase). The degradation of the EGL did not produce major changes in the main systemic parameters of the studied animals. We evaluated the hypothesis that as the EGL determines CFL dynamics, blood flow, and shear rates, it consequently affects blood apparent viscosity. By degrading the EGL, we investigate *in vivo* the non-Newtonian relationship between shear rate and shear stress of blood flow in microvessels to establish the implications of the EGL on vascular resistance. Responses to changes in the integrity of the EGL were studied for individual arterioles using intravital microscopy and capturing high-speed video recordings to measure the CFL thickness and blood flow velocity. *In vivo* blood flow measurements were combined with CFL thickness assessment with sufficient spatial and temporal resolution.

## Materials and methods

### Animal model and tissue preparation

Sprague-Dawley rats (Harlan Laboratories, Livermore, CA) weighing 150–185 g were used to perform the studies. Experiments were approved by the Institutional Animal Care and Use Committee at University of California San Diego and conducted in accordance with the Guide for the Care and Use of Laboratory Animals (National Research Council Committee US, 2010). The cremaster muscle model, for which the complete surgical preparation is elsewhere described in detail, was used for observation of the microcirculation (Baez, 1973). Briefly, 60 mg/kg pentobarbital sodium was injected ip for anesthesia of rats. Throughout the experiment, additional anesthesia was administered as needed. Blood withdrawals and pressure measurements were obtained from the catheterized femoral artery. Catheterization of the right jugular vein was utilized for fluid and anesthesia administration. The animal was positioned on a stage where the cremaster muscle was gently lifted and secured by sutures on a temperature controlled plexiglass pedestal (Physitemp Instruments, Inc. Clifton, NJ). Plastic film was used to cover the exposed muscle (Saran, Dow Corning, Indianapolis, IN). Arteriole responsiveness to a physiological stimulus was tested by topically applying adenosine (10^−4^ M) mixed with suffusate under the polyvinyl film. The experimental procedures were started after 20–30 min after the adenosine was washed away. Non-reactive vessels or vessels not recovering baseline diameter within this time period were excluded from study. Similarly, at the end of the study, adenosine (10^−4^ M) was reapplied and non-reactive vessels, or vessels that had not recovered their diameter prior adenosine application 20 min after washing it, were excluded from study. At the end of the experiment, animals were euthanized.

### Intravital microscopy

The experimental setup consisted of an intravital microscope (Olympus-BX51WI) equipped with a matching long working distance condenser (NA = 0.8, Thorlabs, Newton, NJ). Two magnifications of 2X and 1.5X were installed between the objective (40X, LUMPFL-WIR, NA = 0.8; Olympus) and the high-speed camera, providing a total magnification of 1200X with an equivalent resulting pixel size of 0.125 μm relative to the object plane. A mercury arc lamp (100 W, Walker Instruments, Scottsdale, AZ) was used to illuminate the tissue. A 400-nm interference filter (Spectra Physics, no. 59820) was placed above the condenser in the light path to maximize contrast between blood and the surrounding tissue. A high-speed video camera (Fastcam 1024 PCI, Photron USA), equipped with a one-megapixel chip was used for video recording. Additionally, all videos were recorded between 2,000 and 3,000 frames per second and the camera shutter speed was optimized to obtain the highest quality image possible.

### Experimental protocol

As blood rheology is strongly influenced by hematocrit (Hct), the systemic Hct of all animals included in the study was standardized to 40% Hct via hemodilution when needed. Hemodilution was accomplished by concurrent withdrawal of blood from the arterial catheter and infusion of 5% wt/v human serum albumin solution (ABO Pharmaceuticals, San Diego, CA) using the venous catheter. Vessel diameter was continuously monitored, and video recordings for velocity measurements were collected at selected time points.

### Microvascular diameter and cell free layer (CFL) thickness

The analysis of intensity along a video raster line across the vessel was used to determine the vessel diameter (D), RBC column width, and CFL thickness. Detailed information of the algorithm has been previously described (Kim et al., 2006). The CFL thickness was approximated as the distance between the outer edge of the RBC core and the vessel wall. CFL analysis was performed on both sides of the vessel using a sequence of 1,600 consecutive video frames. Diameter and CFL were measured over a 0.8 s time. Vessel diameter was averaged over this period, and only diameters with standard deviation close to zero were used. Diameter measurements with high standard deviation were discarded and repeated, as diameters were not expected to change over this short period. CFL temporal variation (TV) was defined as the standard deviation of the CFL measurements in an entire image sequence divided by the time elapsed (Kim et al., 2007).

### Blood velocity profiles

The instantaneous blood flow velocities at different points along the radial direction of the micro-vessel were determined from velocity profiles measured using 2D cross correlation. Briefly, images are segmented (10 × 10 pixels), and the displacement of each segment relative to the subsequent image is calculated based on the maximum correlation coefficient, from which cross correlation coefficients are calculated. Velocity was calculated as the longitudinal displacement multiplied by the frame rate (2,000 fps). This approach provides a spatial velocity profile for every set of two consecutive frames and allows for a range of velocity measurements between 0.3 and 250 mm/s. Mean velocity was calculated as the arithmetic mean of the velocity profile. Blood flow (Q) was calculated assuming Poiseuille's flow, as:

(1) $$\begin{array}{r} Q=\frac{\pi D^{4}V}{4} \end{array}$$

where D is the vessel diameter, and V is the mean blood flow velocity.

### Enzymatic degradation of the endothelial glycocalyx

Enzymatic degradation of the glycocalyx in the cremaster muscle is achieved by systemic infusion of specific enzyme cocktail. Specific GAGs were cleaved by the enzymes heparinase III (50 U/mL), chondroitinase ABC (10 U/mL) and hyaluronidase (3,000 U/mL) and were given in bolus doses via the femoral catheter (Cabrales et al., 2007). CFL recordings were made at 5, 20, 40, and 60 min after enzyme treatment and for a sham group at the same timepoints. This treatment was selected as it is reported not to produce any significant changes in the main systemic parameters of the studied animals.

### Estimation of the endothelial glycocalyx thickness

EGL thickness was estimated by subtracting the 99% confidence interval of the CFL in a glass tube, in which only fluid hydrodynamic forces from the 99% confidence interval measured *in vivo*. As the CFL is the sum of the effects of the EGL and the fluid hydrodynamic forces, subtraction of the two distributions will yield an approximation of the thickness of the endothelial glycocalyx. However, because the glycocalyx itself has hydrodynamic forces associated with its presence, it is impossible to exactly determine the thickness of the glycocalyx utilizing this methodology.

### Velocity profiles

Velocity profiles were determined via auto-correlation of high-speed intravital microscopy videos before and after enzymatic degradation of the endothelial glycocalyx, and results were fit to the following model:

(2) $$\begin{array}{r} \upsilon \left(r\right)=\;\upsilon_{max}\left(1-{\left|\frac{r}{R}\right|}^{K}\right)\; \end{array}$$

where v(r) is the spatial velocity profile, vmax is the maximum velocity of the profile, R is the radius of the vessel, and K is the blunting coefficient. Least squared fits were determined using the MATLAB Curve Fitting Toolbox (Mathworks, 2017).

### Statistical analysis

Results are presented as mean ± standard deviation. All statistical calculations and graphics were performed with a commercially available software package (Prism 7.0, GraphPad). To test the normality of the distributions, the D'Agostino and Pearson Normality test was used. Group's comparisons were performed for diameter and CFL thickness data utilizing both ordinary one-way ANOVA. In this case, the assumption of normality in the ANOVA test-statistic was validated via the non-parameterized Geisser Greenhouse test to test for sphericity. As the sphericity metric for all comparisons approached 1, it can be concluded that the ANOVA test statistic is Gaussian, even though the original distributions for CFL thickness were non-Gaussian. Pair-wise comparisons for velocity and flow data were performed with a Mann-Whitney *U*-Test. Variance comparisons were performed utilizing the *F*-test. For all tests, *P* < 0.05 was accepted as statistically significant. Histograms and cumulative distribution functions (CDFs) of the CFL were numerically determined and analyzed at different time points. The maximum likelihood of the CFL thickness was approximated as the inflection point of the CDF. Ninety nine confidence intervals are presented from the point at which *P* = 0.01 to the maximum likelihood determined from the CDF. A Bland Altman analysis was utilized to analyze variations in CFL thickness over time as compared to a glass tube:

(3) $$\begin{array}{r} S\left(x,y\right)=\left(\frac{S_{1}+S_{2}}{2},S_{1}-S_{2}\right)\; \end{array}$$

Where S_1_ is the thickness of the CFL at baseline and after enzymatic degradation, and S_2_ is the thickness of the CFL in a glass tube. All animals passed the Grubbs' test, ensuring that all the measured values at baseline were within a similar population (*P* < 0.05).

## Results

A total of 27 animals (*n* = 27) were entered into the study, two or three arteries were selected in each animal. After application of the systemic and vessel reactivity inclusion criteria, data from 20 animals (*n* = 20) were in included in the results. We found no significant differences in the systemic parameters, confirming that the EGL degradation only had microvascular effects without causing systemic hemodynamic disturbance. Additionally, Table 1 presents the thickness of the CFL after enzymatic treatment and in a Control group over time. As demonstrated in the sham group variations in the CFL thickness were within ±5% of that at baseline, while the CFL thickness decreased by 11.4% 40 min after enzymatic treatment. These results provide sufficient evidence that changes in the thickness of the CFL were likely due to enzymatic treatment and not due to spontaneous variations.

**Table 1:** Thickness of the CFL after enzymatic treatment and control groups.

	**Baseline**	**5 min**	**20 min**	**40 min**	**60 min**
Control (% change from BL)	2.04 ± 0.01 μm (0.0%)	2.13 ± 0.01 μm (+4.6%)	2.11 ± 0.01 μm (+3.4%)	2.06 ± 0.02 μm (+0.8%)	1.98 ± 0.01 μm (−3.1%)
Enzyme treatment (% change from BL)	2.76 ± 0.02 μm (0.0%)	2.93 ± 0.02 μm (+7.8%)	2.50 ± 0.01 μm (−5.8%)	2.42 ± 0.02 μm (−11.4%)	3.22 ± 0.02 μm (+18.4%)

### CFL analysis for a single blood vessel

**Figure 1:**
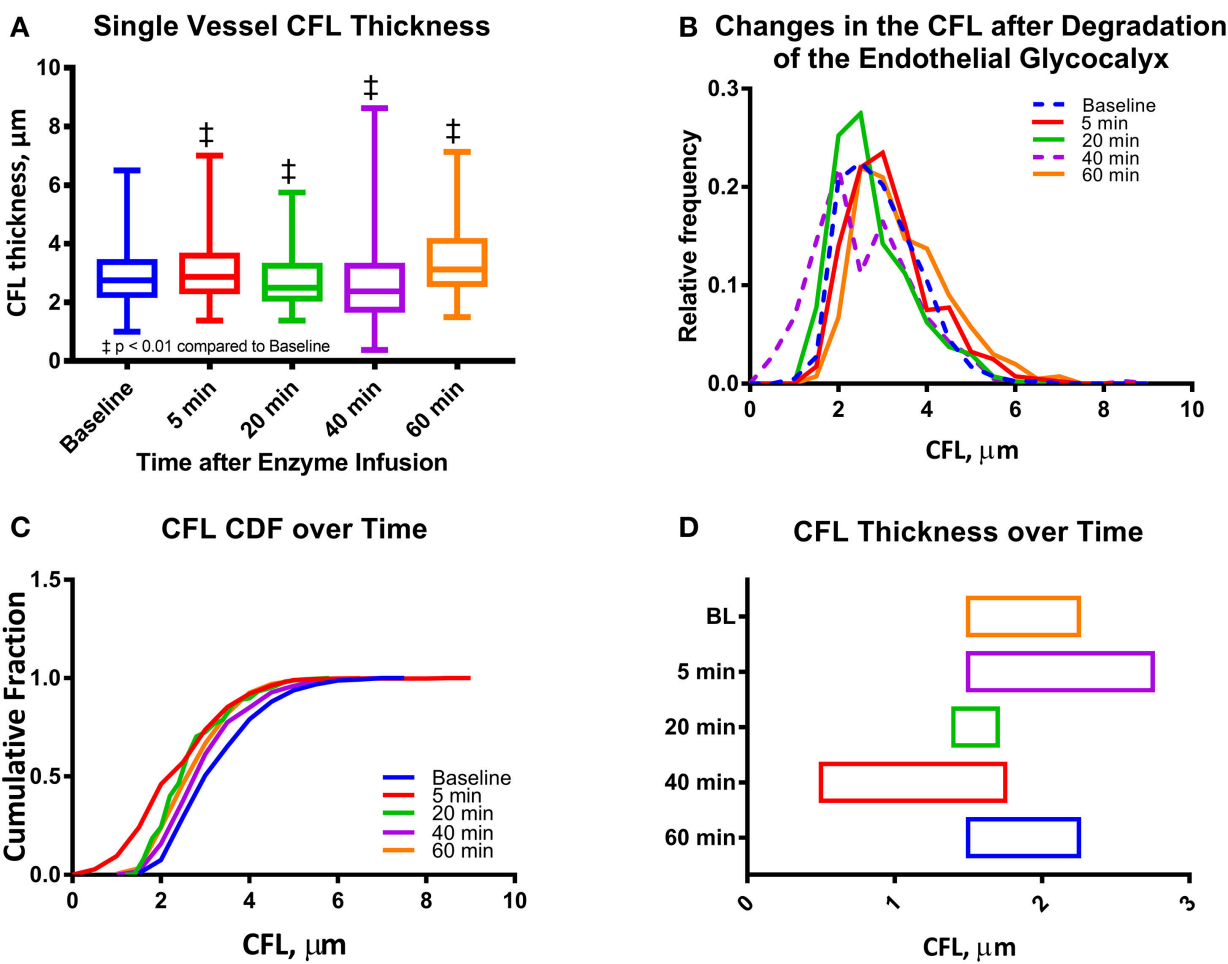
Single microvessel CFL thickness during enzymatic degradation of the EGL: **(A)** CFL thickness over time after enzyme infusion. The changes in the mean CFL were found to be statistically significant (^‡^*P* < 0.05) at all-time points. **(B)** Histograms of CFL thickness over time after enzyme infusion. **(C)** Cumulative distribution functions (CDF) of CFL thickness over time after enzyme infusion. **(D)** One tailed 99% confidence interval of CFL over time after infusion. The left hand bound was determined as the point on the CDF at which *P* < 0.01, and the right hand bound was determined as the inflection point of the CDF (see Supplemental Figure 1). (^‡^*P* < 0.05) compared to baseline.

**Figure 2:**
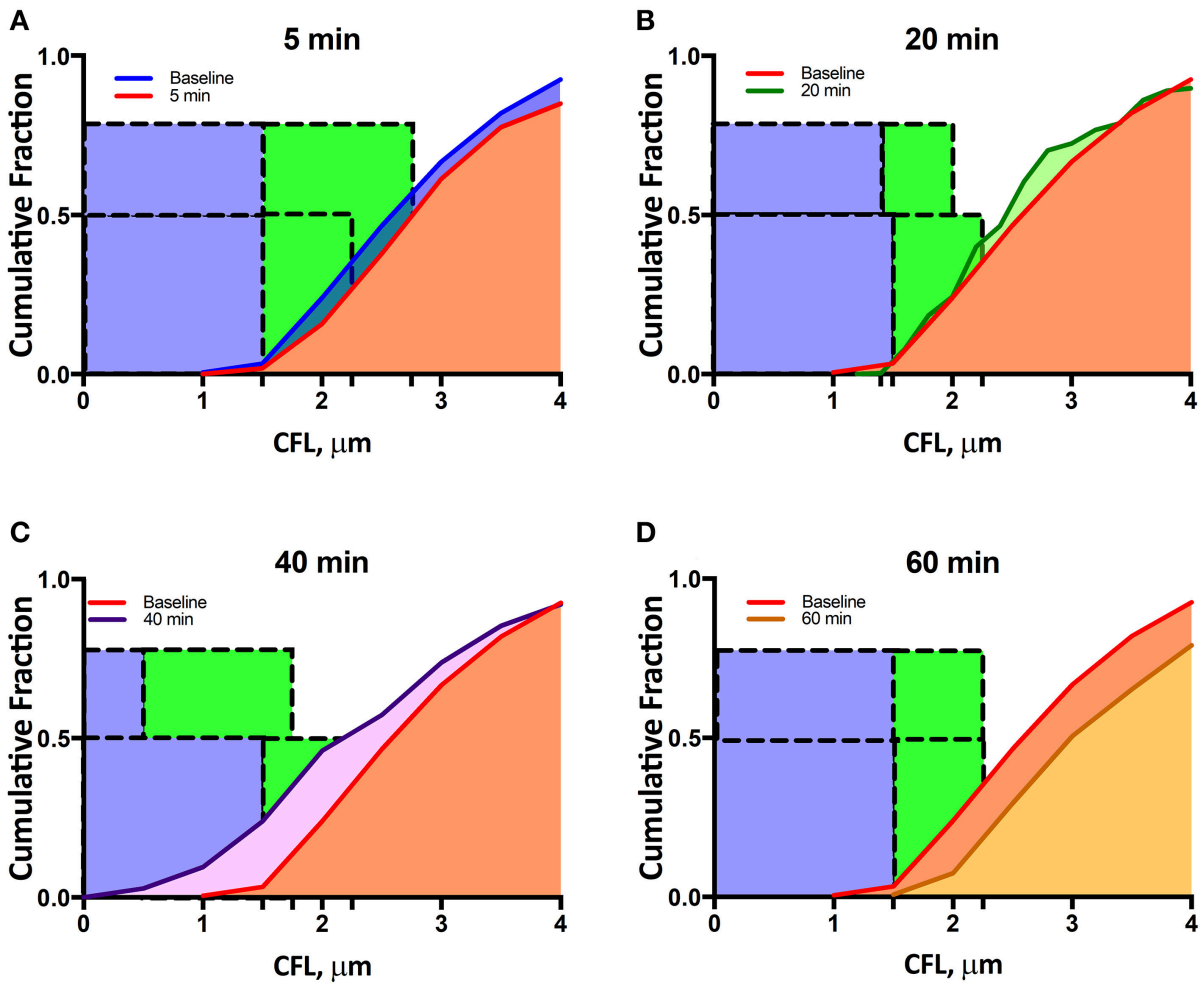
Single microvessel cumulative distribution function during enzymatic degradation of the EGL: CDFs at baseline and at **(A)** 5 min, **(B)** 20 min, **(C)** 40 min, and **(D)** 60 min after enzyme infusion. The blue bonding box represents the portion of the CFL influenced by the glycocalyx and its associated hydrodynamic forces, and the green bounding box represents the one-tailed 99% confidence interval of the CFL thickness (see Supplemental Figure 2).

Initial arteriole diameter was 34–36 μm and maximally decreased to 32–33 μm 20 min after enzymatic degradation of the glycocalyx. No change in diameter was detected in the Control group overtime relative to baseline. The maximal change in diameter measured was <5% from baseline diameter at 20 min after enzymatic degradation of the glycocalyx. The reduction in diameter increases the fraction of the radius of the arterioles occupied by the CFL from 5.8 to 6.1%. Image analysis of high-speed intravital microscopy videos recorded for a single arteriole was quantified in terms of the CFL thickness at different time points after infusion of the enzyme cocktail (Figure 1). Thickness of the CFL is presented in Figure 1A. The CFL thickness was found to be statistically different from baseline at all-time points. The median value of the CFL thickness decreased to 86% of that at baseline at 40 min after enzymatic degradation of the glycocalyx. Distributions of the CFL thickness were also found to deviate from a standard Gaussian to a right-skewed distribution, indicative of the vessel wall acting as a physical boundary to blood flow as the distributions failed the Kolmogorov-Smirnov and the D'Agostino and Pearson Normality Test, *P* < 0.001 (Figures 1B,C). Differences in the variances of the distributions were also found to be statistically significant. The 99% confidence interval of the CFL thickness is presented in Figure 1D. The cumulative fractions of the CFL over time compared to baseline are presented in Figure 2. The 99% confidence interval of the CFL was found to decrease from δ∈[1.50μ*m*, 2.25μ*m*] to a low of δ∈[0.50μ*m*, 1.75μ*m*] at a time of 40 min, where δ is the thickness of the CFL. Additionally, thickness of the CFL increased between 40 and 60 min to δ∈[1.50μ*m*, 2.25μ*m*], indicating recovery of the endothelial glycocalyx.

### CFL analysis from multiple blood vessels

**Figure 3:**
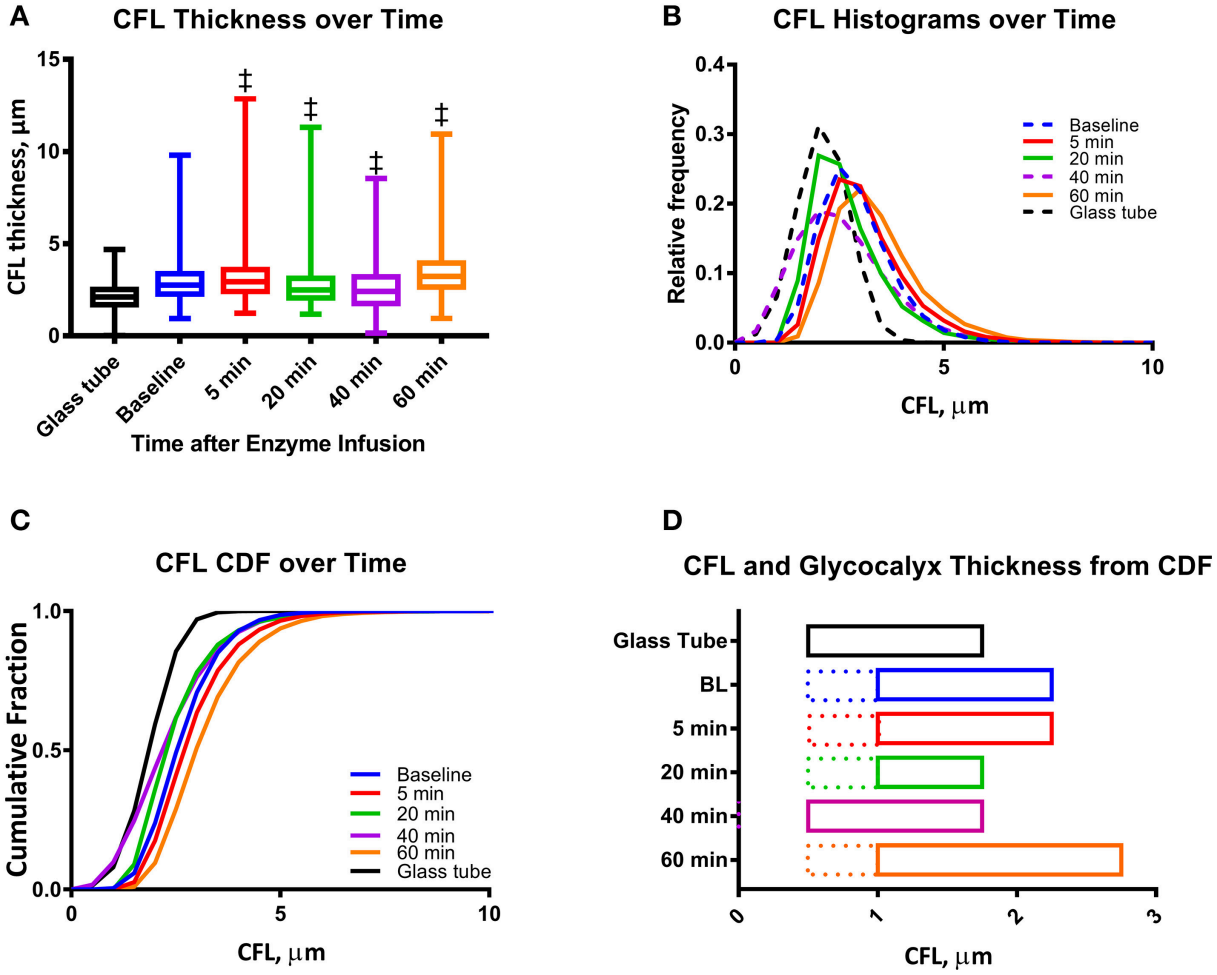
Mean CFL thickness during enzymatic degradation of the EGL: **(A)** CFL thickness over time after enzyme infusion. The changes in the mean CFL were found to be statistically significant (^‡^*P* < 0.05) at all-time points. **(B)** Histograms of CFL thickness over time after enzyme infusion. For individual histograms, see Supplemental Figure 2. **(C)** Cumulative distribution functions (CDF) of CFL thickness over time after enzyme infusion. **(D)** One tailed 99% confidence interval of CFL over time after infusion. The left hand bound was determined as the point on the CDF at which *P* < 0.01, and the right hand bound was determined as the inflection point of the CDF (see Supplemental Figure 1). The thickness of the EGL (indicated as dotted line) was approximated by correcting the CFL distributions with that measured for a glass tube, in which only fluid hydrodynamic forces are present. (^‡^*P* < 0.05) compared to baseline.

**Figure 4:**
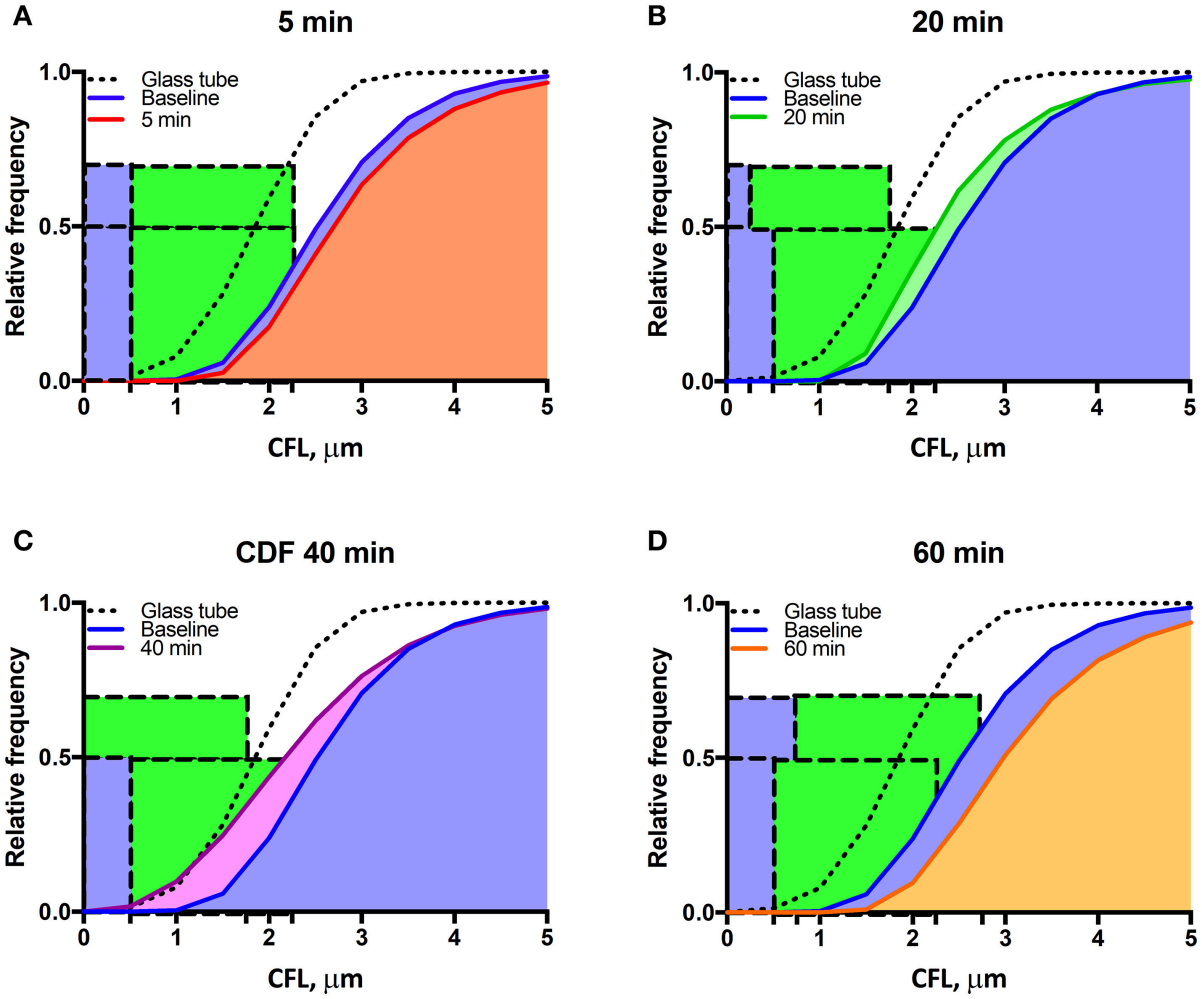
Mean cumulative distribution function during enzymatic degradation of the EGL: CDFs at baseline and at **(A)** 5 min, **(B)** 20 min, **(C)** 40 min, and **(D)** 60 min after enzyme infusion. The blue bonding box represents the approximated thickness of the glycocalyx, and the green bounding box represents the one-tailed 99% confidence interval of the CFL thickness.

Changes in vascular tone for all the 46 arterioles (ranging from 30 to 36 μm) from 20 different animals were not statistically significant at the different time points compared to baseline. The thickness of the CFL for the 46 arterioles is presented in Figure 3A. Similar to the analysis of a single vessel, there were statistically differences in the CFL thickness over all time points compared to baseline. The CFL thickness does not fit a Gaussian (normal) distribution, indicating random variation, as the distributions failed the D'Agostino and Pearson Normality Test, *P* < 0.001. The distribution of the CFL thickness was observed to be right-skewed, which is due to the vessel wall acting as a physical barrier to the thickness of the CFL (Figures 3B,C). The 99% confidence interval of the CFL thickness for 46 arterioles is presented in Figure 3D. The cumulative fractions of the CFL over time compared to baseline are showed in Figure 4. The 99% confidence interval of the CFL was found to decrease from δ∈[1.00μ*m*, 2.25μ*m*] to a low of δ∈[0.50μ*m*, 1.75μ*m*] at a time of 40 min. Additionally, thickness of the CFL increased between 40 and 60 min to of δ∈[1.00μ*m*, 2.75μ*m*]. For reference, the thickness of the CFL in a glass tube was measured and the 99% confidence interval was found to be δ∈[0.50μ*m*, 1.75μ*m*]. In addition, the size of the 99% confidence interval of the CFL was found the statistically larger than that at baseline via the *F*-test, indicating unequal spatial recovery of the EGL after enzymatic degradation.

### EGL thickness

Glycocalyx thickness was found to be approximately 0.50 ± 0.02 μm and was found to degrade to 0.25 ± 0.02 μm 20 min after infusion of the enzyme cocktail (Figures 3A, 4).

### Variances in CFL thickness over time

**Figure 5:**
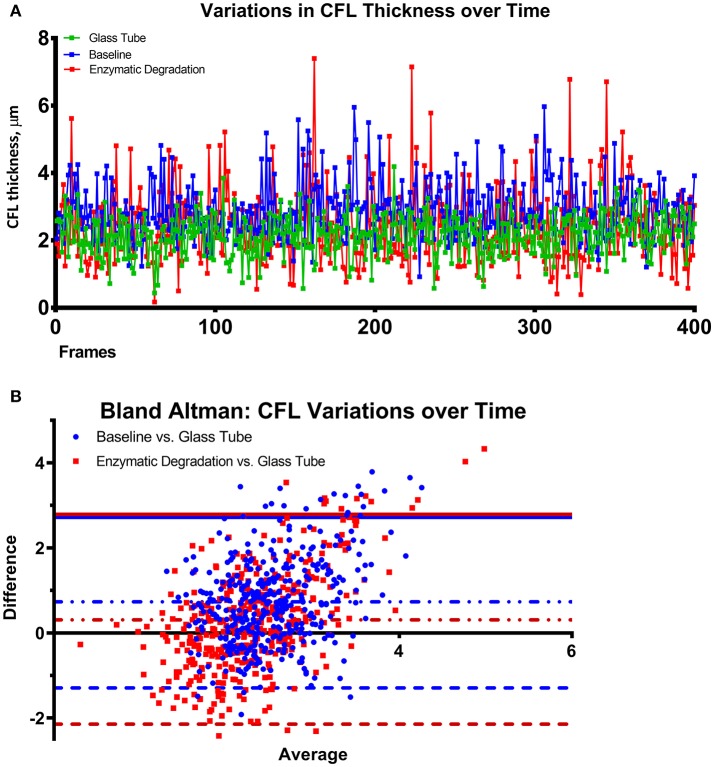
Thickness of the CFL over time after enzymatic degradation of the EGL. **(A)** Fluctuations in the thickness of the CFL over time in a glass tube, at baseline, and after enzymatic degradation of the glycocalyx. **(B)** Bland Altman plots comparing the difference of the CFL thickness vs. a glass tube at baseline and after enzymatic degradation of the glycocalyx. The 95% confidence interval of the difference between the CFL of the blood vessels are illustrated with lines. The solid line indicates the positive end of the difference between the CFL of the blood vessels and the glass tube, which remained unaltered as it is determined by the vessel wall. The dotted line indicates the negative boundary of the difference between the CFL of the blood vessels and the glass tube, illustrates the increased erratic variations in the CFL of the blood vessel after the glycocalyx degradation.

Variations in the CFL thickness over time before and after enzymatic degradation of the CFL are shown in Figure 5A and compared to those in a glass tube of equal diameter in Figure 5B via a Bland-Altman plot. The mean deviation of the CFL from that of a glass tube statistically decreased after enzymatic degradation of the EGL. The size of the 95% confidence interval did not change after degradation of the EGL. The negative end of the 95% confidence interval changed, while the positive end remained constant, determined by the presence of the vessel wall. Comparing the variance of the CFL thickness from the blood vessel to the glass tube before and after enzymatic degradation of the glycocalyx, shows that degradation of glycocalyx increases the difference in the CFL between the blood vessel and the glass tube. The positive end of the difference remained unaltered, because it is indicative of the vessel wall, whereas the negative boundary of the difference shows the increased erratic variations in the CFL of the blood vessel after the degradation of the glycocalyx.

### Blood flow and blood velocity over time

**Figure 6:**
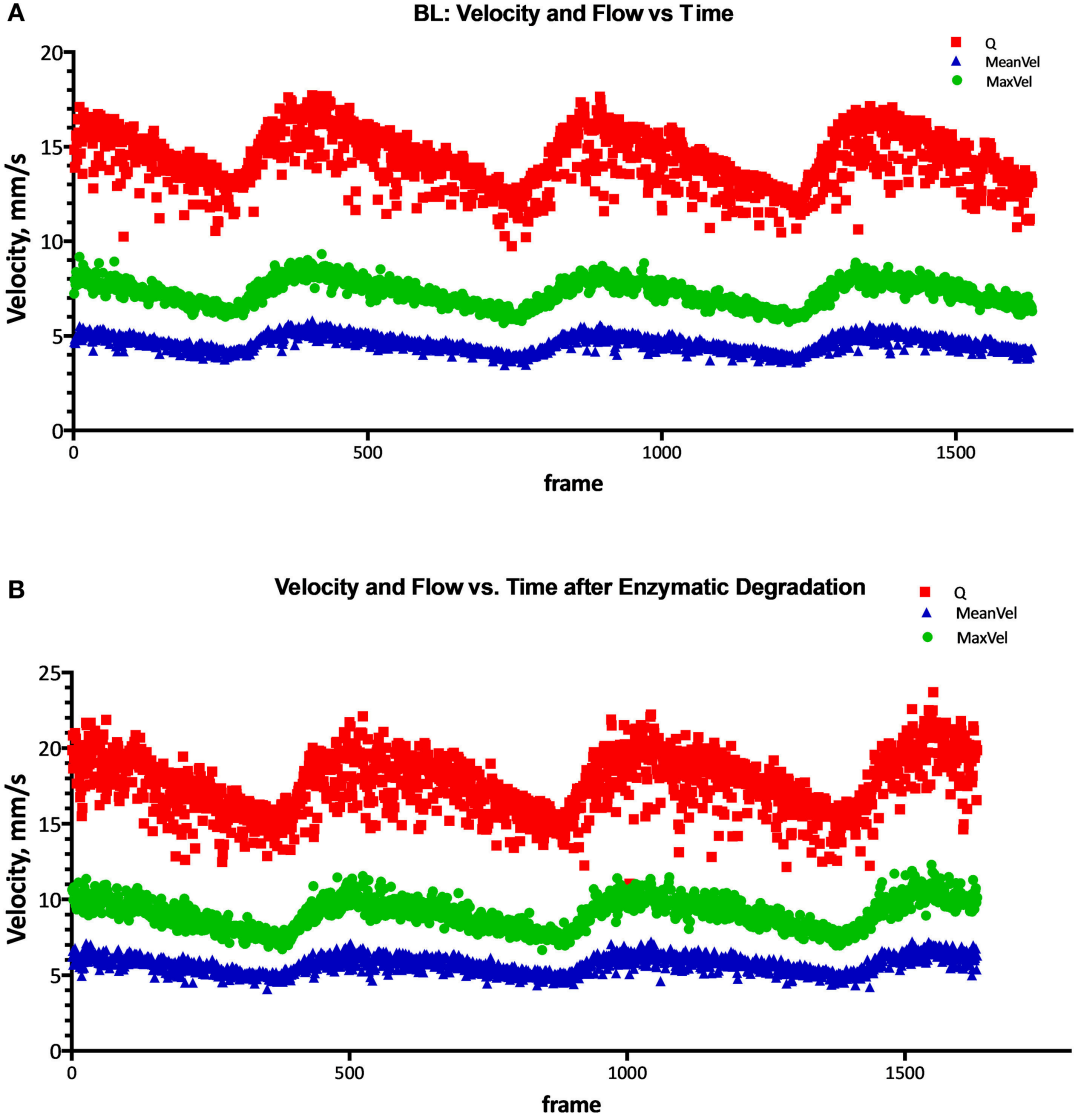
Flow, mean velocity, and peak velocity after degradation of the EGL. Flow (Q), mean velocity (V_mean_), and peak max velocity (V_max_) at **(A)** Baseline, and **(B)** after enzymatic degradation of the glycocalyx.

**Figure 7:**
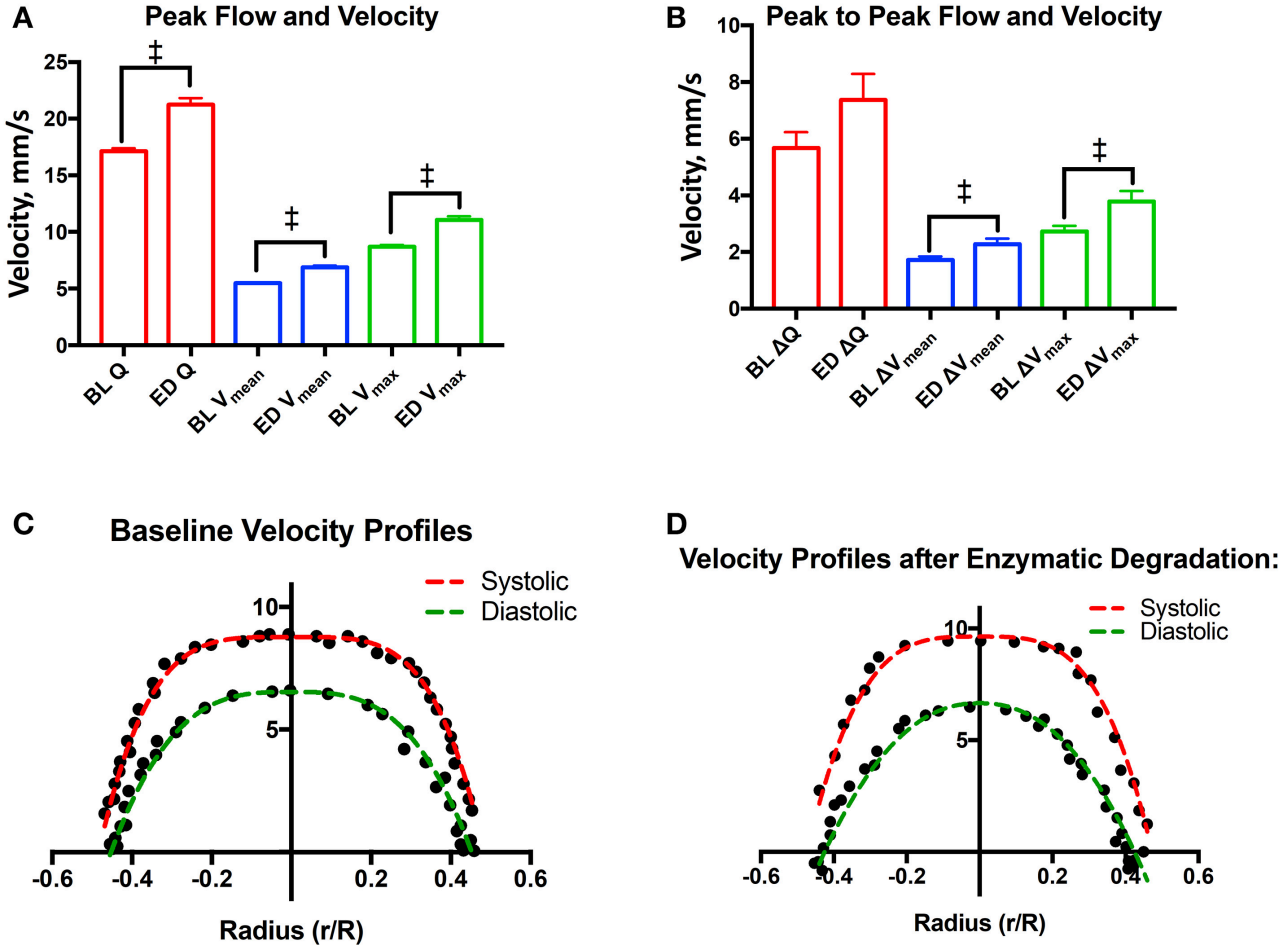
Velocity profiles after degradation of the EGL: **(A)** Systolic flow, mean velocity, and peak velocity after degradation of the glycocalyx (^‡^*P* < 0.05). **(B)** Changes in flow (Q), mean velocity (V_mean_), and max peak velocity (V_max_) between systole and diastole after degradation of the glycocalyx (^‡^*P* < 0.05). **(C)** Baseline velocity profiles during systole and diastole. Results were fit to the model, $\upsilon \left(r\right)=V_{max}\left(1-{\left|\frac{r}{R}\right|}^{K}\right)$. During systole, *V*_*max*_ = 8.8 ± 0.2, *R* = 0.48 ± 0.01, and *K* = 4.1 ± 0.4. During diastole *V*_*max*_ = 6.5 ± 0.4, *R* = 0.45 ± 0.01, and *K* = 3.1 ± 0.6. **(D)** Velocity profiles during systole and diastole after enzymatic degradation of the glycocalyx. Results were fit to the model, $\upsilon \left(r\right)=V_{max}\left(1-{\left|\frac{r}{R}\right|}^{K}\right)$. During systole, *V*_*max*_ = 9.6 ± 0.5, *R* = 0.47 ± 0.01, and *K* = 3.4 ± 0.7. During diastole *V*_*max*_ = 6.7 ± 0.7, *R* = 0.43 ± 0.01, and *K* = 2.2 ± 0.6. (^‡^*P* < 0.05) compared to baseline.

Max blood flow, max blood velocity, and mean velocity were found to statistically increase by 24, 27, and 25%, respectively, after enzymatic degradation of the EGL. Additionally, the change in peak-to-peak max velocity and peak-to-peak mean velocity between were found to statistically increase by 39 and 32%, respectively, after enzymatic degradation of the EGL. However, the increase in the change in peak-to-peak flow was not found to be statistically significant after degradation of the EGL. Results are summarized in Figures 6, 7A,B.

### Velocity profiles after enzymatic degradation of the EGL

Velocity profiles before and after degradation of the EGL are presented in Figures 7C,D. During systole, the blunting coefficient decreased from 4.1 ± 0.4 to 3.5 ± 0.7 after enzymatic degradation of the EGL. Similarly, during diastole, the blunting coefficient decreased from 3.1 ± 0.6 to 2.2 ± 0.6 after enzymatic degradation of the EGL. Thus degradation of the EGL decreased microvessel blood velocity bluntness and a shift toward Poiseuille flow.

## Discussion

The principle finding of this study is that enzymatic degradation of the EGL resulted in a statistically decrease in the CFL thickness, as well as a statistical increase in max blood flow, and max and mean blood velocity. Enzymatic degradation of the EGL also decreased the blunting of the blood velocity profile in arterioles, indicating a shift toward Poiseuille flow. Previous studies have posed models to explain the hydrodynamic and physical forces associated with the EGL (Pries et al., 1990, 1997; Secomb et al., 1998; Long et al., 2004; Weinbaum et al., 2007). Our study provides *in vivo* validation of the hydrodynamic effects of the EGL on blood flow. Changes in the thickness of the CFL during enzymatic degradation compared to the CFL thickness in glass tubes confirms the physical presence of the glycocalyx and the hydrodynamic lubricating layer it produces, and allows for estimation of the apparent thickness (0.5 μm). Literature and mathematical models describe the effects of enzymatic degradation of the EGL on microvascular blood flow as the balance between two opposing forces. As the glycocalyx is a physical barrier to blood flow, it decreases the effective diameter, or the diameter accessible to blood flow, of the lumen. In this schema, degradation of the EGL increases the effective diameter of the lumen, thus decreasing vascular resistance and consequently decreasing blood flow. However, the anionic character of the glycocalyx imparted to it by the glycoproteins and glycosaminoglycans that constitute it are a source of electrical forces that effectively increase the apparent viscosity of blood by forcing negatively charged RBCs toward the center of the lumen, known as the electro-viscous effect. In this schema, degradation of the EGL will decrease the apparent viscosity of blood, increasing blood flow. The effects of the EGL on microvascular blood flow are a summation of both phenomena. As observed in this study, in the case of a 34 μm, blood flow was found to increase with enzymatic degradation of the EGL, indicating that electro-viscous effects are more dominant for arterioles of this size. The thickness of the EGL and ESL are a small fraction of the arteriole lumen diameter, which increases as the arteriole diameter decreases. Therefore, degradation or damages in the EGL have different effects on microvascular blood flow depending on the arteriole diameter, as the electro-viscous effect of the EGL on blood flow becomes less significant in smaller arterioles. The small changes in diameter induced by the enzymatic degradation of the EGL are not responsible for the changes in the CFL thickness or the hemodynamics measured in the study; although, the vasoconstriction induced by the degradation of the EGL is a confounding effect that overestimates the fraction of the arteriole lumen occupied EGL.

The analyzed video recordings determined arteriolar blood flow using spatial cross-correlation analysis, which allows for measurements of blood flow velocities up to 40 mm/s with a precision that ranges between 0.5 and 1 μm (Ortiz et al., 2014) Arteriolar diameters exhibited minimal variations during the experiments after enzyme treatment. As such, arteriolar blood flow velocity was used to estimate relative changes in network flow resistance. Enzymatic degradation of the EGL decreases the plug flow behavior of the blood velocity profile shifting toward pipe flow behavior, as demonstrated by the decreasing magnitude of the blunting coefficient (Figures 7C,D). These results are once again in accordance with the electro-viscous effects produced by the EGL as the electrical forces present in the CFL invalidate the no-slip boundary condition that defines pipe flow. As degradation of the EGL decreases the magnitude of the electrical forces present in the CFL, pipe flow is observed after degradation of the glycocalyx, as the no-slip boundary condition is now valid. This paradigm can also be explained with respect to a classical fluid dynamics model posed by Damiano et al. in which the presence of the EGL promotes Darcy flow in the CFL by increasing the velocity gradients in the plasma interface, thus increasing viscous drag forces in the CFL (Damiano, 1998; Weinbaum et al., 2003). In addition to providing a mechanism for the mechanotransductive behavior, the presence of Darcy flow in the CFL invalidates the no-slip condition, necessary for pipe flow (Weinbaum et al., 2003; Tarbell and Shi, 2013). The EGL is thought to behave as a porous material with a high hydraulic resistance, and consequently plasma velocity through the EGL is very limited; although RBCs essentially slide over the EGL. Thus, degradation of the EGL could increase vascular resistance via preventing RBCs from gliding over the vessel wall.

Enzymatic degradation of the glycocalyx directly affects the ESL, the active composite of bound plasma constituents attached to the EGL. The ESL *per se* influences blood cell-vessel wall interactions, affects blood rheology, and determines solute transport out of the vessel lumen. The ESL function has been suggested to safeguard the fragile EGL from fluid shear stress and from direct physical interactions with blood cells (Secomb et al., 2001). Increased endothelial shear increases nitric oxide (NO) production, dilating vessels and reducing shear stress (Jacob et al., 2007). Additionally, it has been observed that under shear stress, human umbilical vein endothelial cells double the hyaluronic acid in the endothelial glycocalyx, suggesting a secondary mechanism by which the vascular endothelium responds to shear stress (Gouverneur et al., 2006). As the EGL is exposed to the shear stress of the plasma layer, a function of both the shear rate at the surface of the glycocalyx and the plasma viscosity, the surface of the ESL is exposed to the shear stress of the adjacent whole blood. The resultant effect on the signal transduction pathways of the endothelium could then be combined effects of fluid shear stress on both layers. Previous studies on relationship between the ESL in capillaries and flowing blood demonstrate that the ESL can be highly deformable (Long et al., 2004). However, the finite resistance to compression of the ESL prevents direct plasma shear stress to be generated by blood flow on endothelial cells by decreasing plasma fluid movement within the ESL (Pries et al., 2000). The plasma shear stress cannot be transmitted to the ESL as a fluid shear stress; rather, the ESL transfers its shear strain to the structures forming the ESL (Secomb et al., 2001). Enzymatically degrading the EGL impairs transduction of plasma fluid shear stress and solid shear stress in the ESL, which are both potential mechanisms of endothelial shear stress mechanotransduction (Florian et al., 2003). Therefore, endothelial cell surface shear stress transmitted to through the ESL, or plasma fluid shear stress within the ESL, remain plausible as the mechanism of blood flow mediated mechanotransduction by endothelial cells.

In addition, in capillaries, the ESL creates a large repulsive forces that supports the movement of RBCs by acting as a lubrication layer, which reduces capillary blood flow resistance (Secomb et al., 1998; Feng and Weinbaum, 2000). This observation highlights the role of the EGL in microvascular perfusion. Pathophysiological degradation of the EGL would increase capillary blood flow resistance by decreasing the thickness of the lubrication layer provided by the EGL. Recent studies have demonstrated that in chronically obese subjects, diabetic subjects, and subjects with coronary microvascular disease, the EGL is found to be thinner as compared to healthy subjects, resulting in impaired vasomotor activity, increased vascular permeability, and decreased perfusion. Additional studies have demonstrated marked improvement in perfusion in animal models with coronary microvascular disease by increasing recovery of the glycocalyx with metformin and sulodexide (van Haare et al., 2017). Therefore, as the ESL and the glycocalyx decrease capillary blood flow resistance, it seems plausible that pathophysiological degradation of the EGL may play a role in decreased perfusion associated with obesity and related pathophysiologies and influence further progression of these pathophysiologies (van Haare et al., 2017).

Various approaches have been used directly visualize or estimate the EGL due to the increased recognition of its functional importance. Groups have labeled the glycocalyx with specific markers that attach to one or more of its components, making them fluorescent or detectable (Vink and Duling, 1996). This demonstrates the presence of the EGE, or its components, but it does not establish the thickness of the EGL. Unfortunately, the EGL is easily disturbed and very vulnerable to dehydration. Consequently, the EGL dimension is easily underestimated; early studies estimated EGL thickness to be approximated 20 nm in capillaries using transmission electron microscopy (TEM) (Luft, 1966). Other attempts using TEM reported the EGL to be around 40 nm (Ueda et al., 2004). These early estimations did not conform to experimental and theoretical estimation however, suggesting that the EGL should be nearer 1 μm (Klitzman and Duling, 1979; Vink and Duling, 1996). The thickness of the EGL remains controversial, as resent studies with fluorescent labeling and high-resolution fluorescent micro-particle image velocimetry suggest that the GGL is around 0.5 μm in post-capillary venules of the rat mesenteric and the mouse cremaster muscle, both using intravital microscopy (Smith et al., 2003; Gao and Lipowsky, 2010). Recently, new imaging protocols using fluorescently tagged antibodies to heparan sulfate and hyaluronan has revealed a much thicker EGL, over 4.0 μm in the mouse common carotid artery (Megens et al., 2007), 2.2 μm in the mouse internal carotid artery (van den Berg et al., 2009), and 2.5 μm in the external carotid artery (Reitsma et al., 2011). Most recently, cryo-TEM, which avoids the dehydration artifacts of early TEM, has suggested that the thickness of the EGL is of the order of 10 μm on cultured endothelial cells *in vitro* (Ebong et al., 2011). Therefore, the thickness of the EGL measured from our method is in good agreement with both the classical method for measurement of the EGL and other indirect methods (Weinbaum et al., 2007). The approach used in this manuscript to estimate the EGL based on the hydrodynamic implications that the presence of the EGL has in the thickness of the CLF relative to glass tubes of similar diameters. The estimation of the EGL presented in here could differ from the physical thickness of the EGL; although it corresponds to the functional hydrodynamic layer that affect blood flow and exchanges between the flowing blood and the tissues.

The EGL affects blood apparent viscosity in microvessel via the CFL thickness, which is the region with much lower viscosity than the lumen core heavily populated with pack RBCs. Variations in the thickness of the CFL are a consequence of both the Fahraeus effect and the Fahraeus–Lindqvist effect (Fåhraeus, 1929). In the microvasculature (<40 μm), the CFL thickness is significant percentage of the microvessel tube diameter, resulting in smaller microvessels having a lower relative apparent viscosity compared to larger vessels (>40 μm). The effects of the EGL with respect to the Fahraeus effect have an impact on oxygen flux to tissues and NO scavenging by hemoglobin in the RBCs, as the EGL contributes to the separation of RBCs from the vascular endothelium. The separation of blood flow between the vessel lumen core, rich of RBCs, and the endothelium, by the CFL zones affects oxygen delivery and NO consumption by RBCs. Computational models for oxygen and NO transport indicate that the CFL determines resistance to diffusion of oxygen and NO to and from RBCs (Lamkin-Kennard et al., 2004; Ng et al., 2015). In addition, the CFL thickness becomes more important for controlling and enhancing oxygen release and NO bioavailability to both the surrounding tissue and the vascular wall in microvessels with diameters of 30 μm or less (Lamkin-Kennard et al., 2004). The glycocalyx might have additional effects in NO transport, as it modulates vessel wall shear stress-dependent NO production by the endothelium.

The blood flow velocity profiles appear to be a function of the CFL thickness, and therefore in the vessel wall shear stress on the endothelium walls. The thickness of the CFL and its variability is demonstrated by fluctuations in pressure about the mean, as quantified by the standard deviation of the pressure wave. Consequently, changes in CFL thickness counteract changes in blood flow; as CFL thickness increases, volumetric blood flow rate decreases. Volumetric blood flow rate also decreases as the correlation length decreases and/or the standard deviation of the amplitude increases. The mean shear rate can also increase with fluctuations in the endothelium and the CFL. Increasing or decreasing fluctuations in the endothelium can also extend the entrance length (the length through which the flow regime is not fully-developed, and shear stress statistics are constant). Thus, blood viscosity, estimated from *in vivo* experiments by Poiseuille's law, depends not only on the rheological properties of blood, but also by the statistical parameters that quantify endothelium roughness.

The distribution of plasma and RBCs at the vascular wall has traditionally been attributed to the Fahraeus effect and axial migration of red cells (Fåhraeus, 1929). However, it is now well established that ESL also importantly affect this distribution (Pries et al., 2000). The ESL significantly restricts the approach of RBCs and plasma to the vascular wall (Kim et al., 2007). This study was conducted to evaluate the effects of the effects of enzymatic degradation of the EGL on blood flow via preparation of the rat cremaster muscle. We evaluated the hypothesis that enzymatic degradation of the EGL resulted in variations and reductions of the CFL, increased randomness of blood flow, and variations in the observed microhemodynamics. By measuring the change in the thickness of the CFL over time after degradation as well as the probability distribution of RBCs as a function of distance, it is possible to establish a relationship between the micro hemodynamic properties of blood flow and the EGL. Recent studies have demonstrated that the glycocalyx influences homogenous flow distribution in the microcirculation (McClatchey et al., 2016). In addition, many recent studies have implicated the alterations in the mechanotransductive effects of the EGL under different pathophysiological conditions, including inflammation, diabetes, and atherosclerosis (Harrison et al., 2006). As such, future studies should investigate changes in the hemodynamics under these pathophysiological conditions that result due to alterations in the EGL utilizing *in vivo* models.

## Conclusion

These results demonstrate that enzymatic degradation of the EGL results in a statistically significant increase in flow and mean velocity as well as a decrease in the bluntness of the velocity profile in the microcirculation. In addition to providing *in vivo* validation of many mathematical models that explain the hydrodynamic effects of the EGL, these results suggest that the EGL is responsible for alterations in blood flow in microvessels via both the electrical and hydrodynamic fluid forces that it produces. Future studies should investigate the changes in hydrodynamics associated with pathophysiological alterations in the EGL.

## Author contributions

All authors listed have made a substantial, direct and intellectual contribution to the work, and approved it for publication.

### Conflict of interest statement

The authors declare that the research was conducted in the absence of any commercial or financial relationships that could be construed as a potential conflict of interest.

## Supplementary material

Supplemental Figure 1 Numerical differences of the cumulative distribution function of the CFL thickness. To determine the inflection point of the CDF, the second numerical difference of order 4 was determined, and the bisection method was used to determine the root of the second derivative.

Supplemental Figure 2 Mean histograms of the thickness of the CDF. Histograms of the thickness of the CDF during **(A)** Baseline, and **(B)** 40 min after enzyme infusion.
