# Supplementary figures and images for: Implications Enzymatic Degradation of the Endothelial Glycocalyx on the Microvascular Hemodynamics and the Arteriolar Red Cell Free Layer of the Rat Cremaster Muscle
Source: Front Physiol. 2018 Mar 16;9:168. doi: 10.3389/fphys.2018.00168 (PMC5864934; doi:10.3389/fphys.2018.00168)

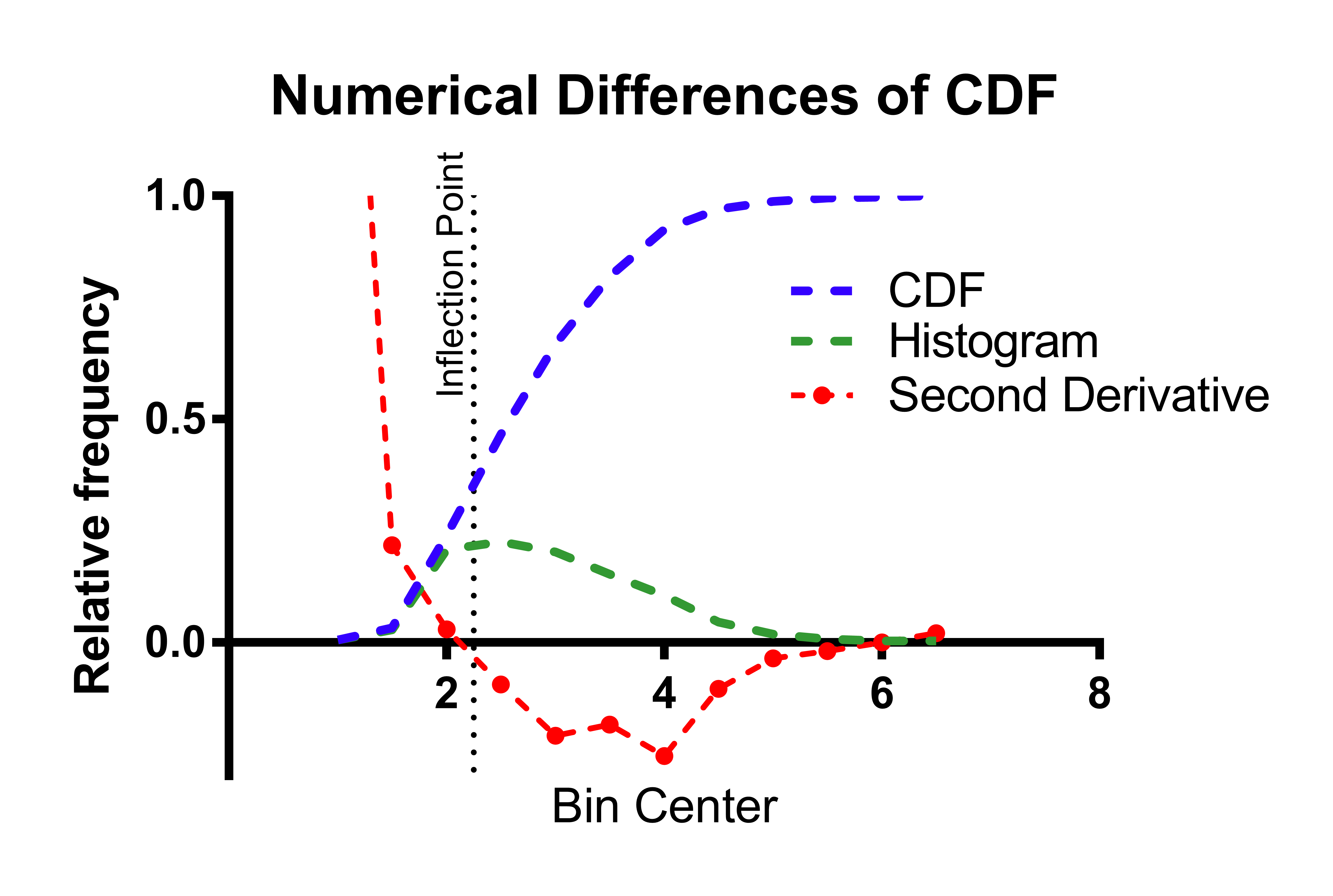

Supplement: Supplemental Figure 1 — Numerical differences of the cumulative distribution function of the CFL thickness. To determine the inflection point of the CDF, the second numerical difference of order 4 was determined, and the bisection method was used to determine the root of the second derivative. [file Image1.tif]

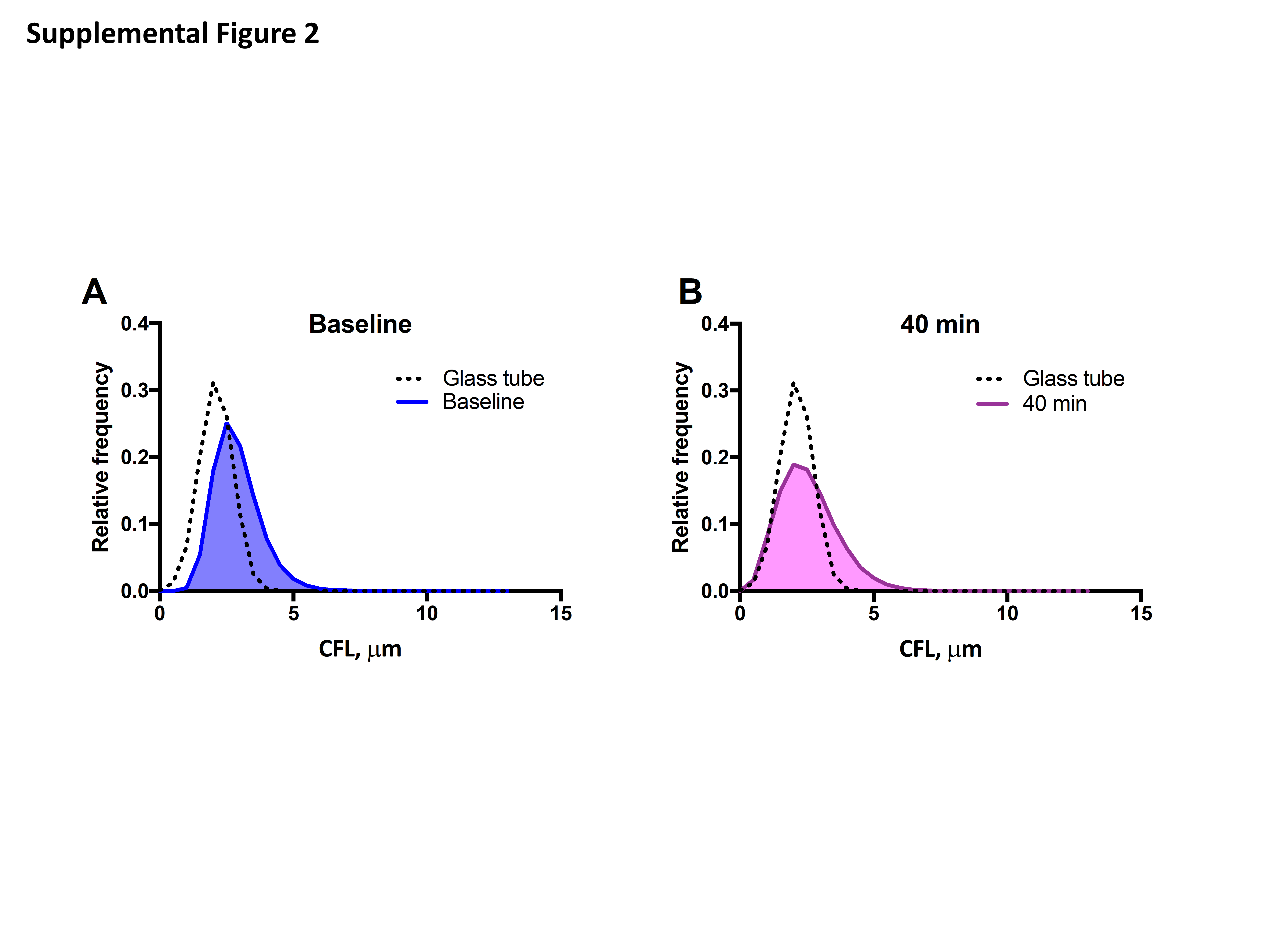

Supplement: Supplemental Figure 2 — Mean histograms of the thickness of the CDF. Histograms of the thickness of the CDF during (A) Baseline, and (B) 40 min after enzyme infusion. [file Image2.TIF]
